# Time and Type of Administered Fluids during Cesarean Section Might Not Matter for Hemodynamic Outcomes, but There Are Significant Patient Safety Concerns Regarding Colloid Use in Parturients. Comment on Theodoraki et al. Colloid Preload versus Crystalloid Co-Load in the Setting of Norepinephrine Infusion during Cesarean Section: Time and Type of Administered Fluids Do Not Matter. *J. Clin. Med.* 2023, *12*, 1333

**DOI:** 10.3390/jcm12144753

**Published:** 2023-07-18

**Authors:** Başak Akça, Federico Bilotta

**Affiliations:** 1 Hacettepe University, School of Medicine, Department of Anaesthesiology and Reanimation, Ankara 06100, Turkey; 2 Department of Anaesthesiology and Reanimation, School of Medicine, Sapienza University of Rome, 00185 Rome, Italy

We read the article by Theodoraki K and colleagues entitled ‘Colloid Preload versus Crystalloid Co-Load in the Setting of Norepinephrine Infusion during Cesarean Section: Time and Type of Administered Fluids Do Not Matter’ with interest [1]. The aim of this study was to compare the incidence of maternal hypotension in parturients receiving either a colloid preload or a crystalloid co-load in the setting of prophylactic norepinephrine infusion during an elective cesarean section under combined spinal–epidural anesthesia. The authors concluded that both fluid-loading techniques are ‘appropriate’ for women undergoing cesarean delivery.

There are some possible related concerns worth being discussed further.

1. The intraoperative use of hydroxyethyl starch (HES) solutions remain controversial regarding that the perioperative use of HES have increasingly been reported to both the US Food and Drugs Administration and the European Medicines Agency (EMA) [2]. Accordingly, the marketing authorization of HES solutions across the EU was suspended on 26 January 2018 by the EMA [3]. Despite this decision, due to the fact that there were some unanswered questions regarding the generalizability of the data, the use of HES remained as a treatment option [4]. This is especially important relevant in procedures like cesarean delivery that are possibly associated with serious periprocedural complications related to bleeding.

2. According to an international consensus statement, maternal hypotension following spinal anesthesia should be treated or prevented routinely with vasopressors [5]. In this statement, it has been concluded that HES solutions should only be used in addition to vasopressors [5]. In the method of the study by Theodoraki K and colleagues, the pre-emptive use of HES in parturients is deeply concerning since HES is a drug known to have the potential of increasing the risk of bleeding, renal injury, and mortality; although, much smaller volumes of HES were used compared to those used in the trials reporting these complications [5].

3. In a study aiming to determine the effects of in vitro hemodilution with HES on the coagulation status of women, it was concluded that an HES solution causes significant hypocoagulable changes in the thromboelastographic profile [6]. From another point of view, obstetric patients undergo many physiological changes affecting hemostasis in addition to congenital/acquired disorders of coagulation [7]. We would very much appreciate if the authors could explain the rationale for using a drug that is a potential anticoagulant on a group of patient undergoing spinal an esthesia who already have coagulation issues, which means taking into account the several complications like epidural hematoma?

4. The safety category of HES during parturiency and breastfeeding is C, which means ‘the effects on fetus is unknown and adverse effects were obtained in animal reproduction studies on the fetus and there are no adequate and well-controlled studies in humans, but potential benefits may warrant use of the drug in pregnant women despite potential risks’. There are not enough data to recommend the use of HES for children, and it is widely accepted that administration of HES to pregnant and breastfeeding women must be limited to emergency cases only [5].

Given the abovementioned concerns and the relevance of coagulation abnormalities that might eventually result into extensive bleeding and a hysterectomy and the limitations provided by EMA, is it worth using HES instead of using vasopressors as the first-line therapy in patients undergoing cesaren delivery without any hemodynamic instability?

We believe that clarification of these issues will result in better deductions and improve the anesthetic management of parturients with safety concerns.
